# Supplementary figures and images for: Collateral effect of COVID-19 on orthopedic and trauma surgery
Source: PLoS One. 2020 Sep 8;15(9):e0238759. doi: 10.1371/journal.pone.0238759 (PMC7478708; doi:10.1371/journal.pone.0238759)

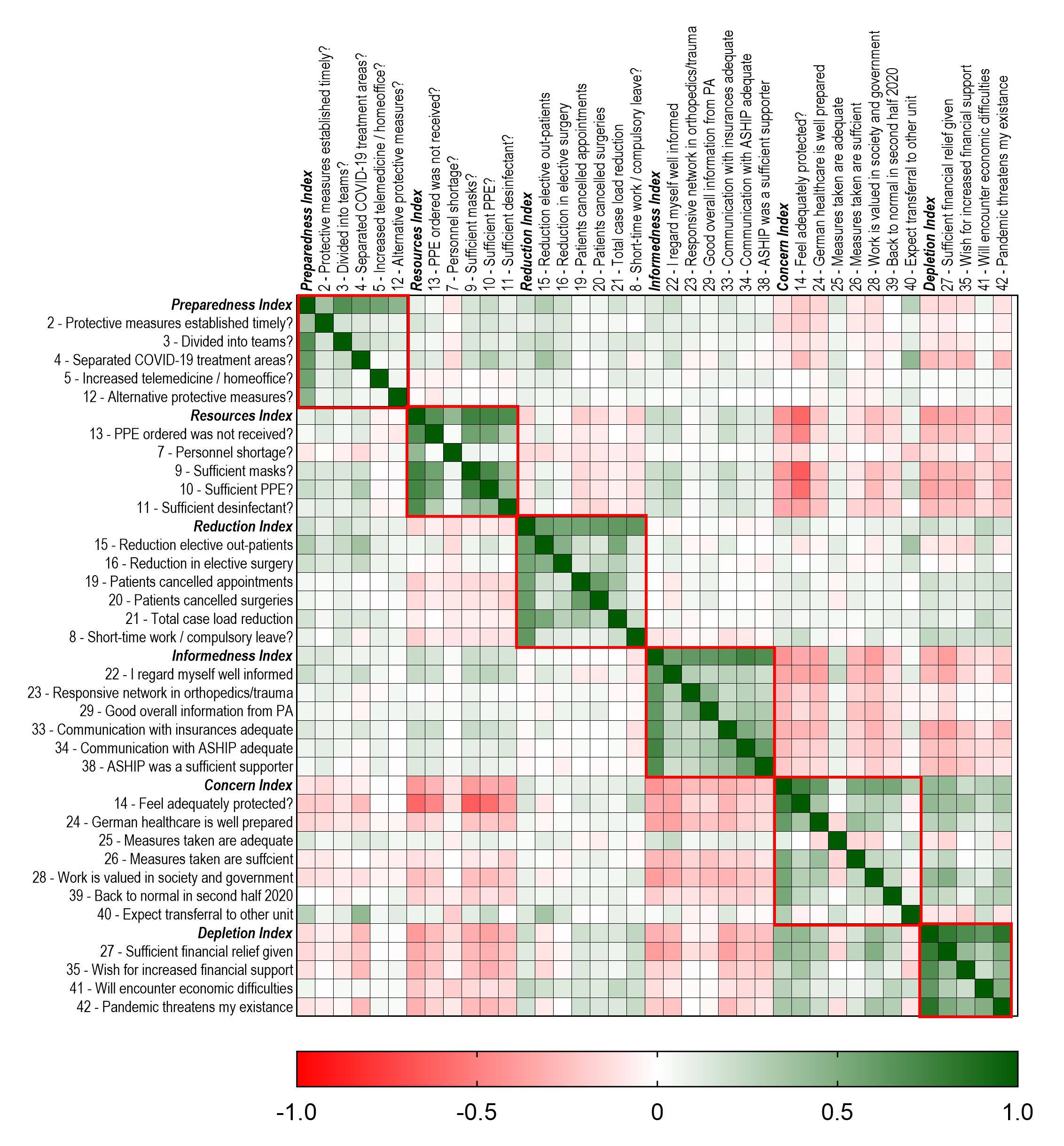

Supplement: S1 Fig — The graph shows a heat map of the spearman correlation between those questionnaire items that were used to calculate the six indices (bold italics). The numbers refer to the item on the questionnaire. For a full list of the indices and the mode of calculation, see S1 Table. Red boxes indicate a negative correlation (Spearman r < 0), green boxes indicate a positive correlation (Spearman r > 0), with darker color representing stronger correlation and white or light colored boxes no or weak correlation. It can be seen how almost all questions assigned into one index correlate very well with the index itself, as well as with the other questions in that index (red frame boxes), as a method of validation for the indices. COVID-19: Coronavirus Disease 19; PPE: Personal protection equipment; PA: Professional association of orthopedics and trauma surgeons; ASHIP: Association of statutory health physicians. (JPG) [file pone.0238759.s001.jpg]

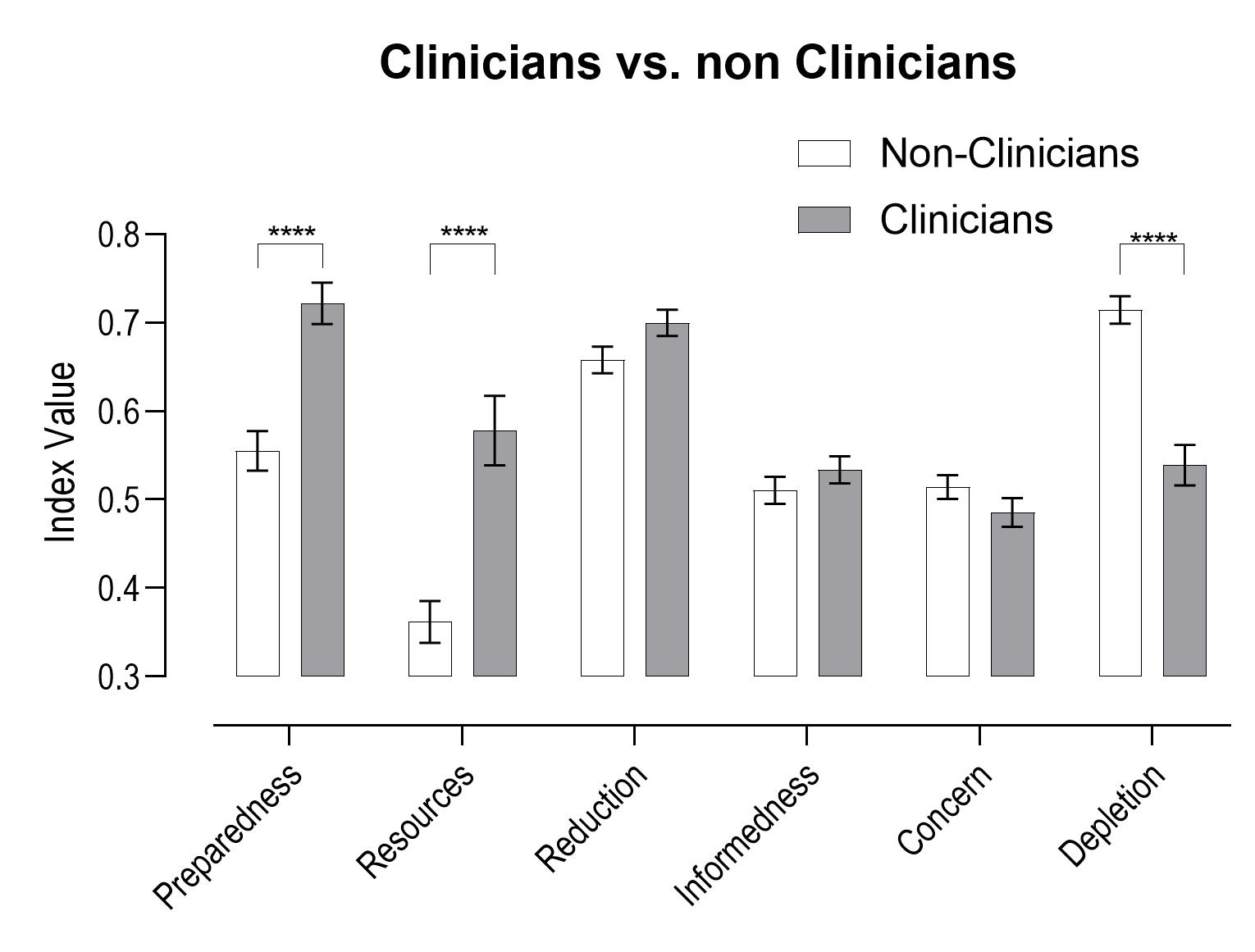

Supplement: S2 Fig — **** indicates an uncorrected P < 0.00038 and thereby significant difference between the groups with manual Bonferroni correction. (JPG) [file pone.0238759.s002.jpg]
