# Supplementary material for: Collateral effect of COVID-19 on orthopedic and trauma surgery
Source: PLoS One. 2020 Sep 8;15(9):e0238759. doi: 10.1371/journal.pone.0238759 (PMC7478708; doi:10.1371/journal.pone.0238759)
Supplement: S1 Table — (DOCX) [file pone.0238759.s003.docx]

**S1 Table:** Composition of the six indices from the questionnaire items and their weighting in the indices

| **Index / Item** | **Possible Value** |
| --- | --- |
| **Preparedness: Mean of the five items below, as a measure of the degree of preparations towards the pandemic (with a range from 0: unprepared to 1: extensive preparation measures taken)** | **Preparedness-Index (0 to 1)** |
| I/my practice /my clinic established protective measures and organizational methods to contain and fight COVID-19 in a timely manner. | Applies (1), Neutral (X), Does not apply (0), Abstain (X) |
| In my institution, the personnel was divided up into smaller teams to minimize risk of infection. |  |
| In my instiution, patients with and without existing COVID-19 tests were isolated in separate treatment areas. |  |
| Due to the COVID-19 pandemic, the use of telemedicine tools / „homeoffice“ by myself and my colleagues has increased. |  |
| I / my practice / my clinic have/has acquired alternative solutions to established protective equipment (Protective screens, selfproduced disinfecting agent,…). |  |
| **Resources: Mean of the five items below, as a measure of the availability of materials and resources for pandemic management (with a range from 0: severe lack of resources to 1: no lack of resources)** | **Resources-Index (0 to 1)** |
| I / my practice / my clinic have/has acquired a sufficient amount of face masks (N95 /FFP2 / FFP3). | Applies (1), Neutral (X), Does not apply (0), Abstain (X) |
| I / my practice / my clinic have/has acquired a sufficient amount of personal protective clothing (protective overalls / gowns / gloves). |  |
| I / my practice / my clinic have/has acquired a sufficient amount of disinfectant. |  |
| Due to quarantine measures or sickness, my institution has expierenced personnel shortages. |  |
| I / my practice / my clinic have/has not acquired sufficient amounts of personal protective equipment despite placing respective orders. | Applies (0), Neutral (X), Does not apply (1), Abstain (X) |
| **Reduction: Mean of the six items below, as a degree of case load reduction in the practice/clinic (with a range from 0: no reduction to 1: near complete shut-down of non-emergency surgery)** | **Reduction-Index (0 to 1)** |
| The treatment of elective patients in O & TS (out-patient clinic) has been reduced in my institution by: | 80-100 (1), 60-80 (0.75), 40-60 (0.5), 20-40 (0.25), 0-20 (0), Abstain (X) |
| Elective surgical procedures in O & TS have been reduced in my institution by: |  |
| The percentage of patients, who themselves cancelled their scheduled ambulant appointments, amounts to: |  |
| The percentage of patients, who themselves cancelled their sheduled surgical procedures, amounts to: |  |
| Due to the Covid-19 pandemic, the number of patients in my institution has been reduced by: |  |
| Due to the COVID-19 pandemic, employees in my institution were ordered to short-time work, compulsory leave and the reduction of overtime. | Applies (1), Neutral (X), Does not apply (0), Abstain (X) |
| **Informed: Mean of the six items below, as a measure of the self-perceived level of being informed about the pandemic and its consequences (with a range from 0: feeling poorly informed to 1: feeling very well informed)** | **Informedness Index (0 to 1)** |
| I regard myself as well-informed in regards to current regulations and measures concerning COVID-19. | Fully agree (1), rather agree (0.75), Neutral (0.5), Rather disagree (0.25), Fully disagree (0), Abstain (X) |
| There exists a cooperating network between O&TS practices and clinics to actively respond to the COVID-19 pandemic. |  |
| The professional association and – society alltogether exhibit a good performance in response to the COVID-19 pandemic. |  |
| The communication with health insurance providers concerning the COVID-19 pandemic is appropriate. |  |
| The communication with the Association of Statutory Health Insurance Physicians in regard to the COVID-19 pandemic is appropriate. |  |
| I experience the KV as a supportive institution for physicians in the current situation in O & TS. |  |
| **Concern: Mean of the seven items below, as a measure of the self-perceived level of being informed about the pandemic and its consequences (with a range from 0: feeling poorly informed to 1: feeling very well informed)** | **Concern Index (0 to 1)** |
| I feel that my colleagues and I are adequately protected in regards to COVID-19 in my current work environement. | Applies (0), Neutral (X), Does not apply (1), Abstain (X) |
| The German healthcare system in general is well-prepared in regard to the COVID-19 pandemic. | Fully agree (0), rather agree (0.25), Neutral (0.5), Rather disagree (0.75), Fully disagree (1), Abstain (X) |
| The measures introduced in the fight against the COVID-19 pandemic so far are sufficient. |  |
| Our medical practice in O & TS is valued by our government and society even in the context of the ongoing pandemic. |  |
| I predict that the mode of operation in my institution will normalize in the second half of 2020. |  |
| The measures introduced in the fight against the COVID-19 pandemic so far are necessary. | Fully agree (1), rather agree (0.75), Neutral (0.5), Rather disagree (0.25), Fully disagree (0), Abstain (X) |
| I expect that I will be transferred to perform medical work outside of my own specialty (e.g. intensive care unit, treatment of ventilated patients, emergency treatment, …) |  |
| **Concern: Mean of the four items below, as a measure of the fearof financial depletion and threat to financial security (with a range from 0: having no feel of financial depletion to 1: feeling one’s financial income is at high risk)** | **Concern Index (0 to 1)** |
| In my opinion, the measures taken to provide financial relief for the economic consequences of the COVID-19 pandemic so far are sufficient. | Fully agree (0), rather agree (0.25), Neutral (0.5), Rather disagree (0.75), Fully disagree (1), Abstain (X) |
| I would wish for increases in confirmations of financial aid / security by the ASHIP. | Fully agree (1), rather agree (0.75), Neutral (0.5), Rather disagree (0.25), Fully disagree (0), Abstain (X) |
| I / my practice / my clinic will encounter financial difficulties due to the COVID-19 pandemic. |  |
| The COVID-19 pandemic and consecutive regulations and measures are threaten by bare existence. |  |
