# Supplementary material for: Collateral effect of COVID-19 on orthopedic and trauma surgery
Source: PLoS One. 2020 Sep 8;15(9):e0238759. doi: 10.1371/journal.pone.0238759 (PMC7478708; doi:10.1371/journal.pone.0238759)
Supplement: S2 Table — (DOCX) [file pone.0238759.s004.docx]

**S2 Table.**

| **Gender** |  |
| --- | --- |
| Female | 113 |
| Male | 738 |
| Diverse | 3 |
| Abstain | 4 |
| **Age Group:** |  |
| < 35 years old | 39 |
| 35-49 years old | 260 |
| 50-69 years old | 546 |
| > 70 years old | 8 |
| Abstain | 5 |
| **Professional level** |  |
| Resident | 38 |
| Consultant | 65 |
| Attending / Chair | 286 |
| Self employed | 457 |
| Other | 9 |
| Abstain | 3 |
