# Supplementary material for: Collateral effect of COVID-19 on orthopedic and trauma surgery
Source: PLoS One. 2020 Sep 8;15(9):e0238759. doi: 10.1371/journal.pone.0238759 (PMC7478708; doi:10.1371/journal.pone.0238759)
Supplement: S1 Data — (DOCX) [file pone.0238759.s005.docx]

A Survey of the Clinic for Orthopedics and Trauma Surgery - University Hospital Bonn and the Center for Musculoskeletal Surgery – Charité University Medicine Berlin:

The **P**erceived **I**mpact of the **C**ovid-19 Pandemic on **O**rthopedic and Trauma **S**urgery in Germany – Survey (PICOS)

| What impact did the COVID-19 Pandemic already have on your practice / clinic / institution | Does apply | Neutral/  unsure | Does not apply | Abstain |
| --- | --- | --- | --- | --- |
| 1. I/my practice /my clinic am/is currently already involved in the treatment of SARS-COV-2 positive patients. |  |  |  |  |
| 1. I/my practice /my clinic established protective measures and organizational methods to contain and fight COVID-19 in a timely manner. |  |  |  |  |
| 1. In my institution, the personnel was divided up into smaller teams to minimize risk of infection. |  |  |  |  |
| 1. In my instiution, patients with and without existing COVID-19 tests were isolated in separate treatment areas. |  |  |  |  |
| 1. Due to the COVID-19 pandemic, the use of telemedicine tools / „homeoffice“ by myself and my colleagues has increased. |  |  |  |  |
| 1. I think telemedicine tools should be applied more frequently in the future. |  |  |  |  |
| 1. Due to quarantine measures or sickness, my institution has expierenced personnel shortages. |  |  |  |  |
| 1. Due to the COVID-19 pandemic, employees in my institution were ordered to short-time work, compulsory leave and the reduction of overtime. |  |  |  |  |

| Which of the following statements about the supply of personal protective equipment applies to you / your institution? | Does apply | Neutral/  unsure | Does not apply | Abstain |
| --- | --- | --- | --- | --- |
| 1. I / my practice / my clinic have/has acquired a sufficient amount of face masks (N95 /FFP2 / FFP3). |  |  |  |  |
| 1. I / my practice / my clinic have/has acquired a sufficient amount of personal protective clothing (protective overalls / gowns / gloves). |  |  |  |  |
| 1. I / my practice / my clinic have/has acquired a sufficient amount of disinfectant. |  |  |  |  |
| 1. I / my practice / my clinic have/has acquired alternative solutions to established protective equipment (Protective screens, selfproduced disinfecting agent,…). |  |  |  |  |
| 1. I / my practice / my clinic have/has not acquired sufficient amounts of personal protective equipment despite placing respective orders. |  |  |  |  |
| 1. I feel that my colleagues and I are adequately protected in regards to COVID-19 in my current work environement. |  |  |  |  |

| Which impact do you experience on your profession / patients? | 80-100 % | 60-80% | 40-60% | 20-40% | 0-20% | Abstain |
| --- | --- | --- | --- | --- | --- | --- |
| 1. The treatment of elective patients in O & TS (out-patient clinic) has been reduced in my institution by: |  |  |  |  |  |  |
| 1. Elective surgical procedures in O & TS have been reduced in my institution by: |  |  |  |  |  |  |
| 1. The treatment of emergency patients in O & TS (emergency room) has been reduced in my institution by: |  |  |  |  |  |  |
| 1. Urgent/Emergency surgical procedures in O & TS have been reduced in my institution by: |  |  |  |  |  |  |
| 1. The percentage of patients, who themselves cancelled their scheduled ambulant appointments, amounts to: |  |  |  |  |  |  |
| 1. The percentage of patients, who themselves cancelled their sheduled surgical procedures, amounts to: |  |  |  |  |  |  |
| 1. Due to the Covid-19 pandemic, the number of patients in my institution has been reduced by: |  |  |  |  |  |  |

| How do you respond – if applicable - to the following statements regarding measures taken by the government and the society as a whole? | Fully agree | Rather agree | Neutral | Rather disagree | Fully disagree | Abstain |
| --- | --- | --- | --- | --- | --- | --- |
| 1. I regard myself as well-informed in regards to current regulations and measures concerning COVID-19. |  |  |  |  |  |  |
| 1. There exists a cooperating network between O&TS practices and clinics to actively respond to the COVID-19 pandemic. |  |  |  |  |  |  |
| 1. The German healthcare system in general is well-prepared in regard to the COVID-19 pandemic. |  |  |  |  |  |  |
| 1. The measures introduced in the fight against the COVID-19 pandemic so far are necessary. |  |  |  |  |  |  |
| 1. The measures introduced in the fight against the COVID-19 pandemic so far are sufficient. |  |  |  |  |  |  |
| 1. In my opinion, the measures taken to provide financial relief for the economic consequences of the COVID-19 pandemic so far are sufficient. |  |  |  |  |  |  |
| 1. Our medical practice in O & TS is valued by our government and society even in the context of the ongoing pandemic. |  |  |  |  |  |  |

| How do you respond – if applicable - to the following statements regarding the performance of the professional association (BVOU) and – society (DGOU) of O & TS? | Fully agree | Rather agree | Neutral | Rather disagree | Fully disagree | Abstain |
| --- | --- | --- | --- | --- | --- | --- |
| 1. The professional association and – society alltogether exhibit a good performance in response to the COVID-19 pandemic. |  |  |  |  |  |  |
| 1. The information policy and support of the professional association and –society was adequate on the federal level. |  |  |  |  |  |  |
| 1. The information policy and support of the professional association and –society was adequate on the state level. |  |  |  |  |  |  |
| 1. The information policy and support of the professional association and –society was adequate on the local level. |  |  |  |  |  |  |

| How do you respond – if applicable – to the following statements regarding measures taken by the Association of Statutory Health Insurance Physicians (KV) and health insurance providers? | Fully agree | Rather agree | Neutral | Rather disagree | Fully disagree | Abstain |
| --- | --- | --- | --- | --- | --- | --- |
| 1. The communication with health insurance providers concerning the COVID-19 pandemic is appropriate. |  |  |  |  |  |  |
| 1. The communication with the Association of Statutory Health Insurance Physicians (ASHIP) in regard to the COVID-19 pandemic is appropriate. |  |  |  |  |  |  |
| 1. I would wish for increases in confirmations of financial aid / security by the ASHIP. |  |  |  |  |  |  |
| 1. The ASHIP supported me / my institution in securing personal protective equipment. |  |  |  |  |  |  |
| 1. The ASHIP facilitated the utilization of telemedicine tools (e.g. video consultations,…) |  |  |  |  |  |  |
| 1. I experience the ASHIP as a supportive institution for physicians in the current situation in O & TS. |  |  |  |  |  |  |

| How do you respond to the following predictions of the upcoming developments regarding the COVID-19 pandemic? | Fully agree | Rather agree | Neutral | Rather disagree | Fully disagree | Abstain |
| --- | --- | --- | --- | --- | --- | --- |
| 1. I predict that the mode of operation in my institution will normalize in the second half of 2020. |  |  |  |  |  |  |
| 1. I expect that I will be transferred to perform medical work outside of my own specialty (e.g. intensive care unit, treatment of ventilated patients, emergency treatment, …) |  |  |  |  |  |  |
| 1. I / my practice / my clinic will encounter financial difficulties due to the COVID-19 pandemic. |  |  |  |  |  |  |
| 1. The COVID-19 pandemic and consecutive regulations and measures are threaten by bare existence. |  |  |  |  |  |  |

| 1. At what kind of institution within O & TS do you currently perform (the majority of) your work? (Only select one answer) |  |
| --- | --- |
| Basic and standard care clinic |  |
| Specialist care clinic |  |
| Clinic of maximum care / University Clinic |  |
| Rehabilitation clinic |  |
| Practitioner in an individual practice |  |
| Practitioner in a shared practice (< 3 physicians) |  |
| Practitioner in a shared practice (≥ 3 physicians) |  |
| Medical occupation without patient care (research, medical service of the health insurance companies, medical expert) |  |
| other |  |
| abstain |  |

| 1. What position do you currently hold in your (predominant) institution. (Only select one answer) |  |
| --- | --- |
| Resident |  |
| Consultant |  |
| Attending doctor, head of department, department chairman |  |
| Self-employed doctor in individual / shared practice |  |
| other |  |
| abstain |  |

| 1. Your patient base consists of: |  |
| --- | --- |
| Exclusivly statutory insured patients |  |
| Statutory and privatly insured patients (usual ratio) |  |
| Mostly privatly insured patients, selectively statutory insured patients |  |
| For the most part privatly insured patients / exclusivly privatly insured patients |  |
| abstain |  |

| 1. Your focus of care is (multiple answers): |  |
| --- | --- |
| Conservative Orthopedics |  |
| Outpatient surgery in an individual / shared practice |  |
| General Trauma Surgery |  |
| Specialized Trauma Surgery – Treatment of critically insured patients (VAV/SAV) |  |
| Joint Surgery / Arthroplasty (including external consultant) |  |
| Spinal Surgery (including external consultant) |  |
| Pediatric orthopedics |  |
| Tumor orthopedics |  |
| Hand and/or Foot Surgery |  |
| Reconstructive and Plastic Surgery |  |
| other |  |
| abstain |  |

| 1. Are you a member in any of the following professional associations? |  |
| --- | --- |
| BVOU – Professional Association of O & TS |  |
| DGOU – German Society Society of O & TS |  |
| DGOOC – German Society of Orthopaedics and Orthopaedic Surgery |  |
| DGU – German Socierty of Trauma Surgery |  |
| None of the above |  |
| Abstain |  |

| 1. What age group do you belong to? |  |
| --- | --- |
| < 35 years |  |
| 35-49 years |  |
| 50-69 years |  |
| > 70 years |  |
| Abstain |  |

| 1. What is your gender? |  |
| --- | --- |
| Female |  |
| Male |  |
| Diverse |  |
| Abstain |  |

1. What else can we do for you? Please provide any additional comments or concerns below:
