# Supplementary material for: Collateral effect of COVID-19 on orthopedic and trauma surgery
Source: PLoS One. 2020 Sep 8;15(9):e0238759. doi: 10.1371/journal.pone.0238759 (PMC7478708; doi:10.1371/journal.pone.0238759)
Supplement: S2 Data — (DOCX) [file pone.0238759.s006.docx]

*Eine Umfrage der Klinik und Poliklinik für Orthopädie und Unfallchirurgie der Universitätsklinik Bonn und des Centrums für Muskuloskeletale Chirurgie - Charité Universitätsmedizin Berlin:*

zur aktuellen Einschätzung der Auswirkungen der COVID-19-Pandemie auf Orthopädie und Unfallchirurgie in Deutschland

The **P**erceived **I**mpact of the **C**ovid-19 Pandemics on **O**rthopedics and Trauma Surgery in Germany – **S**urvey (PICOS)

Sehr geehrte Frau Kollegin, sehr geehrter Herr Kollege!

Wir danken Ihnen für Ihre Teilnahme. Am einfachsten füllen Sie diesen Fragebogen **direkt online** über das sichere Umfrage-Portal der Universität Bonn aus:

[**https://www.covid-ou.de**](https://www.covid-ou.de/)

Alternativ können sie diesen Fragebogen am PC ausfüllen und als DOC oder PDF per Email versenden an [thomas.randau@ukbonn.de](mailto:thomas.randau@ukbonn.de), oder auch den Bogen ausdrucken und postalisch auf den Weg schicken an: Dr. med. Thomas Randau, Uniklinik Bonn, Orthopädie und Unfallchirurgie, Venusberg-Campus 1, 53127 Bonn

Sollten Sie Rückfragen haben, so zögern Sie bitte nicht, uns zu kontaktieren:

PD Dr. med. Matthias Pumberger: [matthias.pumberger@charite.de](mailto:matthias.pumberger@charite.de)

Tel.: 030-450 652156

Dr. med. Thomas Randau: [thomas.randau@ukbonn.de](mailto:thomas.randau@ukbonn.de)

Tel.: 0228-287 14460

**Zum Datenschutz:** Die Erfassung und Verarbeitung aller Daten ist anonym, bitte verzichten Sie daher auf alle Markierungen, die auf Ihre Identität oder oder die Identiät Ihrer Einrichtung schliessen lassen. Alle Ihre Angaben werden streng vertraulich und entsprechend den gesetzlichen Bestimmungen zum Datenschutz behandelt. Alle erhobenen Daten werden nur anonymisiert veröffentlicht und ausschließlich zusammengefasst mit den Angaben der anderen Teilnehmerinnen und Teilnehmer ausgewertet. Rückschlüsse auf Sie persönlich werden nicht möglich sein.

| **Welche Auswirkungen hat die COVID-19 Pandemie bereits auf Ihre eigene Praxis / Klinik / Arbeitseinrichtung?** | **Trifft zu** | **Neutral / weiß nicht** | **Trifft nicht zu** |
| --- | --- | --- | --- |
| 1. Ich/meine Praxis/mein Krankenhaus ist zu diesem Zeitpunkt bereits unmittelbar an der Versorgung von SARS-CoV-2 positiven Patienten beteiligt. |  |  |  |
| 1. Ich/meine Praxis/mein Krankenhaus hat früh und rechtzeitig Schutzvorkehrungen und organisatorische Maßnahmen zur Eindämmung und Bekämpfung von COVID-19 ergriffen. |  |  |  |
| 1. In meiner Einrichtung wurde das Personal in separate Teams aufgeteilt, um das Infektrisiko zu minimieren. |  |  |  |
| 1. In meiner Einrichtung ist eine Trennung in separate Behandlungsbereiche für Patienten mit und ohne Virusnachweis (SARS-CoV-2) erfolgt. |  |  |  |
| 1. Ich arbeite bedingt durch die COVID-19 Pandemie mehr als vorher mit telemedizinischen Verfahren / „HomeOffice“ für mich und meine Mitarbeiter. |  |  |  |
| 1. Ich halte telemedizinische Verfahren für ein Modell, dass auch in Zukunft häufiger angewendet werden sollte. |  |  |  |
| 1. Durch Quarantäne od. Erkrankung kommt es in meiner Einrichtung zu Engpässen beim Personal. |  |  |  |
| 1. Durch die COVID-19-Pandemie wurden in meiner Einrichtung Mitarbeiter in Kurzarbeit, Zwangsurlaub oder Überstundenabbau geschickt oder Mitarbeiter entlassen. |  |  |  |

| **Welche Aussagen zur Versorgung mit persönlicher Schutzausrüstung trifft für Sie / Ihre Einrichtung zu?** | **Trifft zu** | **Neutral / weiß nicht** | **Trifft nicht zu** |
| --- | --- | --- | --- |
| 1. Ich/meine Praxis/mein Krankenhaus hat ausreichend Masken (MNS / FFP2 / FFP3) vorrätig. |  |  |  |
| 1. Ich/meine Praxis/mein Krankenhaus hat ausreichend Körperschutzkleidung (Schutzanzüge/Schutzkittel/ Handschuhe) vorrätig. |  |  |  |
| 1. Ich/meine Praxis/mein Krankenhaus hat ausreichend Desinfektionsmittel vorrätig. |  |  |  |
| 1. Ich/meine Praxis/mein Krankenhaus hat Alternativen zur üblichen Schutzausrüstung etabliert (z.B Schutzscheiben, selbsthergest. Desinfektion/Schutzausrüstung, ..). |  |  |  |
| 1. Ich/meine Praxis/mein Krankenhaus hat trotz Bestellung keine oder nicht ausreichend Schutzausrüstung erhalten. |  |  |  |
| 1. Ich fühle mich und meine Mitarbeiter in meiner aktuellen Arbeitsumgebung bezogen auf COVID-19 gut geschützt. |  |  |  |

| **Welche Auswirkungen sehen Sie bereits auf Ihren Fachbereich / Ihre eigenen Patienten** | **81->100%** | **61-80%** | **41-60%** | **21-40%** | **0-20%** |
| --- | --- | --- | --- | --- | --- |
| 1. Die Behandlung von elektiven Patienten in O&U (Sprechstunden, Terminambulanz) wurde in meiner Einrichtung reduziert um: |  |  |  |  |  |
| 1. Elektive operative Eingriffe in O&U wurden in meiner Einrichtung reduziert um. |  |  |  |  |  |
| 1. Die Behandlung von ambulanten Notfallpatienten aus O&U ist in meiner Einrichtung reduziert um: |  |  |  |  |  |
| 1. Die Durchführung von dringenden / Notfall-OPs in O&U ist in meiner Einrichtung reduziert um: |  |  |  |  |  |
| 1. Der Anteil von Patienten, die selbstständig ihre ambulanten Termine abgesagt haben liegt in meiner Einrichtung bei: |  |  |  |  |  |
| 1. Der Anteil von Patienten, die selbstständig eine geplante Operation abgesagt haben, liegt in meiner Einrichtung bei: |  |  |  |  |  |
| 1. Die Menge an insgesamt zu versorgenden Patienten in meiner Praxis / meinem eigenen Fachbereich hat sich durch COVID-19 reduziert: |  |  |  |  |  |

| **Wie bewerten Sie die folgenden Aussagen zu den Maßnahmen in Politik und Gesellschaft insgesamt?** | **Stimme voll zu** | **Stimme eher zu** | **Neutral** | **Lehne eher ab** | **Lehne völlig ab** |
| --- | --- | --- | --- | --- | --- |
| 1. Ich fühle mich über aktuelle Regulationen und Maßnahmen bezogen auf COVID-19 gut informiert. |  |  |  |  |  |
| 1. In O&U existiert ein kooperierendes Netzwerk zwischen Praxen und Kliniken, um der COVID-19 Pandemie aktiv zu begegnen |  |  |  |  |  |
| 1. Das Gesundheitssystem in Deutschland hat sich insgesamt gut auf die COVID-19 Pandemie vorbereitet. |  |  |  |  |  |
| 1. Die bisher eingeführten Maßnahmen zur Bekämpfung der COVID-19 Pandemie in der Gesellschaft sind notwendig |  |  |  |  |  |
| 1. Die bisher eingeführten Maßnahmen zur Bekämpfung der COVID-19 Pandemie in der Gesellschaft sind ausreichend |  |  |  |  |  |
| 1. Die bisher getroffenen Maßnahmen zur finanziellen Absicherung / Abfederung wirtschaftlicher Folgen empfinde ich für mich persönlich als ausreichend |  |  |  |  |  |
| 1. Auch während der Pandemie wird unsere ärztliche Arbeit im Bereich O&U von Politik und Gesellschaft wertgeschätzt |  |  |  |  |  |

| **Wie bewerten Sie – falls zutreffend - die Arbeit des Berufsverbandes und der Fachgesellschaft?** | **Stimme voll zu** | **Stimme eher zu** | **Neutral** | **Lehne eher ab** | **Lehne völlig ab** |
| --- | --- | --- | --- | --- | --- |
| 1. Berufsverband / Fachgesellschaft leisten insgesamt, bezogen auf die COVID-19 Pandemie, gute Arbeit. |  |  |  |  |  |
| 1. Die Informationspolitik und Unterstützung des BVOU / der DGOU auf Bundesebene war adäquat. |  |  |  |  |  |
| 1. Die Informationspolitik und Unterstützung des BVOU auf Landessebene war adäquat. |  |  |  |  |  |
| 1. Die Informationspolitik und Unterstützung des BVOU auf Bezirksebene war adäquat. |  |  |  |  |  |

| **Wie bewerten Sie – falls zutreffend - die Maßnahmen der kassenärztlichen Vereinigung (KV) und der Krankenkassen?** | **Stimme voll zu** | **Stimme eher zu** | **Neutral** | **Lehne eher ab** | **Lehne völlig ab** |
| --- | --- | --- | --- | --- | --- |
| 1. Die Kommunikation mit den Krankenkassen ist, bezogen auf die COVID-19-Pandemie, angemessen. |  |  |  |  |  |
| 1. Die Kommunikation mit den KV ist, bezogen auf die COVID-19-Pandemie, angemessen. |  |  |  |  |  |
| 1. Ich wünsche mir mehr Zusagen zur finanziellen Unterstützung / Absicherung seitens meiner KV |  |  |  |  |  |
| 1. Die KV hat mich/meine Einrichtung bei der Beschaffung von Schutzausrüstung unterstützt. |  |  |  |  |  |
| 1. Die KV hat die Nutzung von Telemedizin ermöglicht (z.B. Videosprechstunden) |  |  |  |  |  |
| 1. Ich nehme die KV in der aktuellen Situation als Unterstützer der orthopädisch/unfallchirurgischen Fachrichtung wahr. |  |  |  |  |  |

| **Geben Sie uns eine Einschätzung Ihres Ausblicks auf die Zukunft:** | **Stimme voll zu** | **Stimme eher zu** | **Neutral** | **Lehne eher ab** | **Lehne völlig ab** |
| --- | --- | --- | --- | --- | --- |
| 1. Ich schätze, dass sich der Betrieb in meiner Einrichtung ab dem zweiten Halbjahr 2020 wieder normalisiert hat |  |  |  |  |  |
| 1. Ich rechne damit, innerhalb der nächsten Wochen mit fachfremder ärztlicher Tätigkeit betraut zu werden (z.B.: Intensivstation, Betreuung von Beatmungspatienten, Notfallversorgung, o.ä.) |  |  |  |  |  |
| 1. Ich/meine Praxis/mein Krankenhaus wird durch die COVID-19 Pandemie in wirtschaftliche Schwierigkeiten geraten |  |  |  |  |  |
| 1. Die Pandemie und die damit einhergehenden Maßnahmen sind für mich persönlich existenzbedrohend |  |  |  |  |  |

| **Sie sind Mitglied.. (Mehrfachnennung möglich)** | |
| --- | --- |
| im BVOU – Berufsverband für Orthopädie und Unfallchirurgie |  |
| In der DGOU – Deutsche Gesellschaft für Orthopädie und Unfallchirurgie |  |
| In der DGOOC – Deutschen Gesellschaft für Orthopädie und orthop. Chirurgie |  |
| In der DGU – Deutschen Gesellschaft für Unfallchirurgie |  |
| Keine der genannten |  |

| **In welcher Art Einrichtung sind sie aktuell (überwiegend) tätig? (Bitte eine Antwort)** | |
| --- | --- |
| Klinik der Grund- und Regelversorgung |  |
| Klinik mit Schwerpunktversorgung |  |
| Klinik der Maximalversorgung / Universitätsklinikum |  |
| Reha-Klinik |  |
| Niedergelassen in Einzelpraxis |  |
| Niedergelassen in Gemeinschaftspraxis (weniger als drei Ärzte) |  |
| Niedergelassen in Gemeinschaftspraxis (drei oder mehr Ärzte) |  |
| Ärztl. Tätigkeit ohne Patientenversorgung (Forschung, MDK, Gutachter, etc.) |  |
| Sonstiges |  |
| Bitte nennen Sie (optional) die ersten beiden Ziffern der PLZ ihrer Einrichtung: |  |

| **In Welcher Position sind Sie aktuell (überwiegend) tätig? (Bitte eine Antwort)** | |
| --- | --- |
| Assistenzärztin/-arzt bzw. Weiterbildungsassistent/-in in der Praxis |  |
| Fachärztin/Facharzt, FOÄ/FOA bzw. angestellte Fachärztin/-arzt in der Praxis |  |
| Oberärztin/Oberarzt,Sektionsleiter/-in, Chefärztin/Chefarzt |  |
| Selbständig niedergelassen in eigener Praxis / Gemeinschaftspraxis |  |
| Sonstiges |  |

| **Die Patientenversorgung Ihrer Praxis / Klinik / Einrichtung betrifft:** | |
| --- | --- |
| Ausschließlich gesetzlich versicherte (GKV) Patienten |  |
| GKV- und privat versicherte (PKV)-Patienten im allgemein üblichen Mix |  |
| Großteils PKV-Patienten, selektiv auch GKV-Patienten |  |
| Zum überwiegenden Anteil / ausschließlich PKV-Patienten |  |

| **Ihr Versorgungsschwerpunkt liegt auf (multiple Nennungen möglich)** | |
| --- | --- |
| Konservative Orthopädie |  |
| Ambulantes Operieren in der eigenen Praxis |  |
| Allgemeine Unfallchirurgie |  |
| Spezielle Unfallchirurgie mit VAV-/SAV-Versorgung |  |
| Gelenkchirugie / Endoprothetik, auch als Belegarzt in der Klinik |  |
| Wirbelsäulenchirurgie, auch als Belegarzt in der Klinik |  |
| Kinderorthopädie |  |
| Tumororthopädie |  |
| Hand- und/oder Fußchirurgie |  |
| Rekonstruktive und plast. Chirurgie |  |
| Sonstiges / Andere |  |

| **Zu welcher Altersgruppe zählen Sie?** | |
| --- | --- |
| < 35 Jahre |  |
| 35-49 Jahre |  |
| 50-69 Jahre |  |
| > 70 Jahre |  |

| **Welches Geschlecht haben Sie?** | |
| --- | --- |
| Weiblich |  |
| Männlich |  |
| Divers |  |

| **Was können wir (DGOU und BVOU) in diesen Zeiten der Krise noch für Sie tun?** |
| --- |
| Klicken oder tippen Sie hier, um Text einzugeben. |

Vielen Dank für Ihre Teilnahme.

Sofern Sie die Beantwortung auf dem Bogen der Online-Variante auf

<https://www.umfragen.uni-bonn.de/covid> vorgezogen haben, speichern Sie bitte das ausgefüllte Formular als *.docx oder als *.pdf und senden Sie es mit dem Betreff „PICOS-Studie“ per Email an [thomas.randau@ukbonn.de](mailto:thomas.randau@ukbonn.de); Das Formular wird getrennt von Ihrer Email gespeichert; Ihre Email-Adresse wird nicht gesondert gespeichert oder für andere Zwecke verwendet. Sie können uns selbstverständlich auch einen ausgedruckten Bogen postalisch zusenden unter oben angegebener Adresse.
