# Supplementary material for: Collateral effect of COVID-19 on orthopedic and trauma surgery
Source: PLoS One. 2020 Sep 8;15(9):e0238759. doi: 10.1371/journal.pone.0238759 (PMC7478708; doi:10.1371/journal.pone.0238759)
Supplement: S3 Data — (PDF) [file pone.0238759.s007.pdf]

The Percieved Impact of COVID-19 on orhtopedics and trauma surgery:

Sample Size and selction bias

1 – Correlation to the statistics of the federal medical association (Bundesärztekammer)

The federal medical association published some statistical data on the demographic data of physicians registered in Germany on its homepage[1]. From this, we have extracted distribution of age, gender and mode of employment (hospital vs. private practice) of all physicians registered in orthopedics and trauma surgery as available, to compare to our sample:

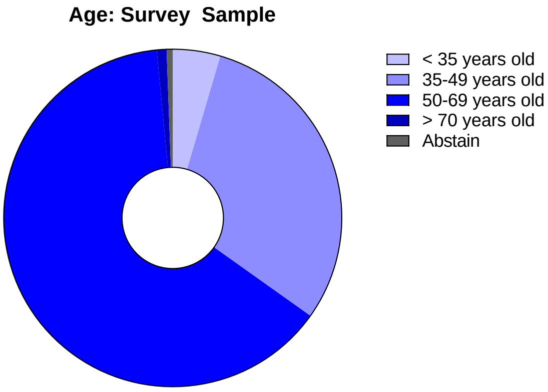

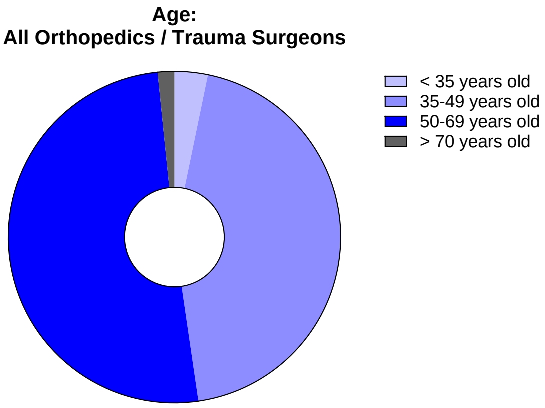

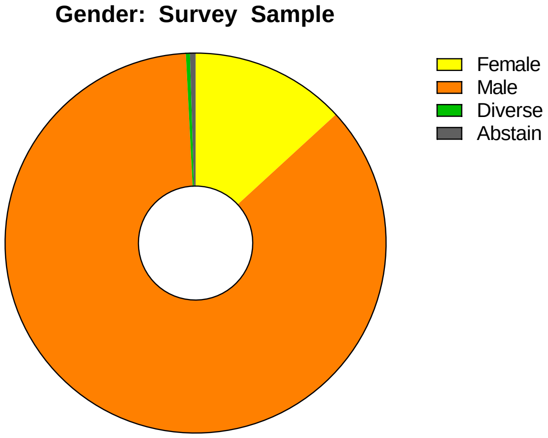

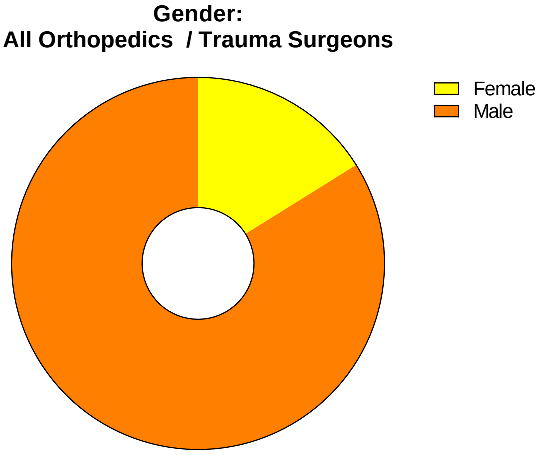

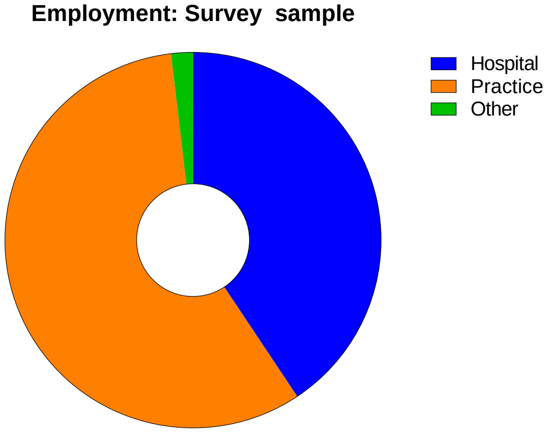

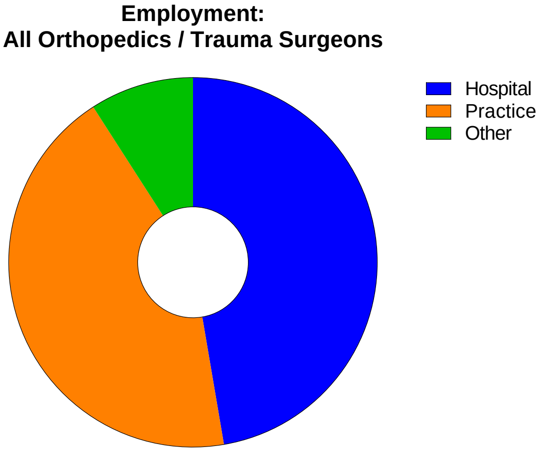

For a sample to be representative, the sample should have similar composition as the whole population. The graphs demonstrate that the survey sample indeed presents a very similar composition. It is not a proportional sample, but we see the risk of a bias due to the small differences to be moderate to low.

---

[1] https://www.bundesaerztekammer.de/ueber-uns/aerztestatistik/aerztestatistik-2019/

2. Second survey sample

The manuscript presents the results of 858 surveys that were fully completed within the two weeks' time of April 2nd to April 16th of 2020. But the online survey was accessed a total of 1785 times without being completed, making these entries "non-participants". Most of the incomplete sets do not contain any data. 176 data sets are available that completed some parts of the survey, and of those, 91 surveys have entries in at least the first two blocks (Items 1 to 14, Figure 1A of the Manuscript). This data was not included in the analysis and can serve as a random second sample

Second Sample Results to items 1-14:

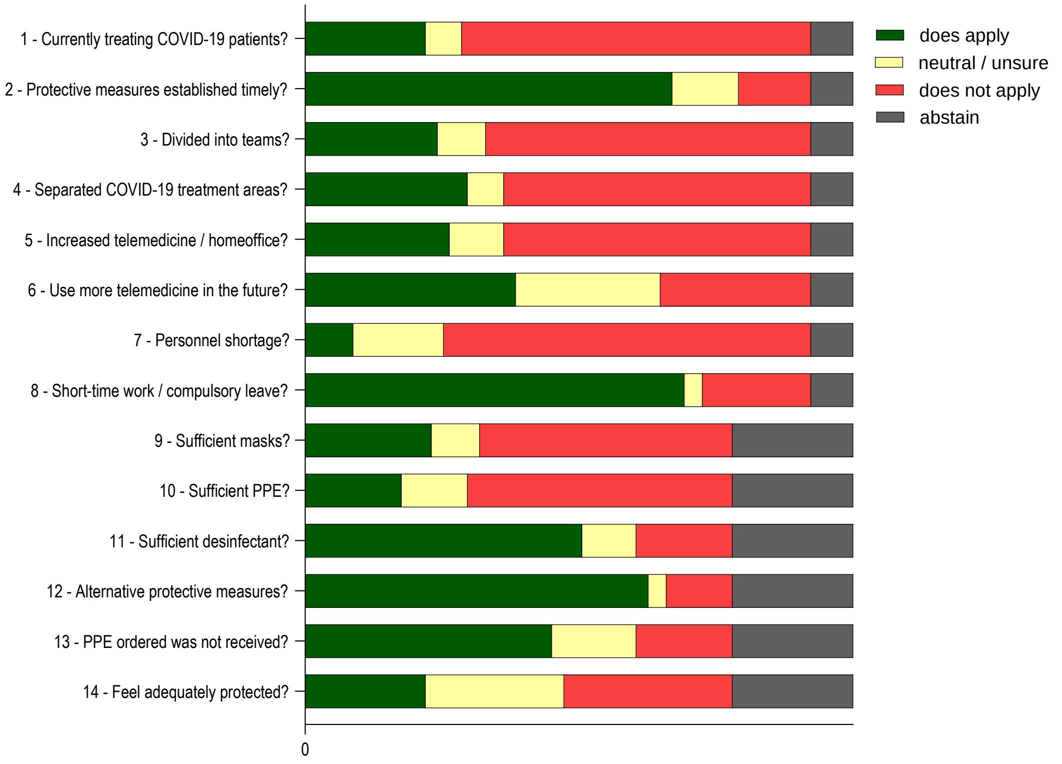

Survey results to items 1-14 (see Figure 1A of the manuscript)

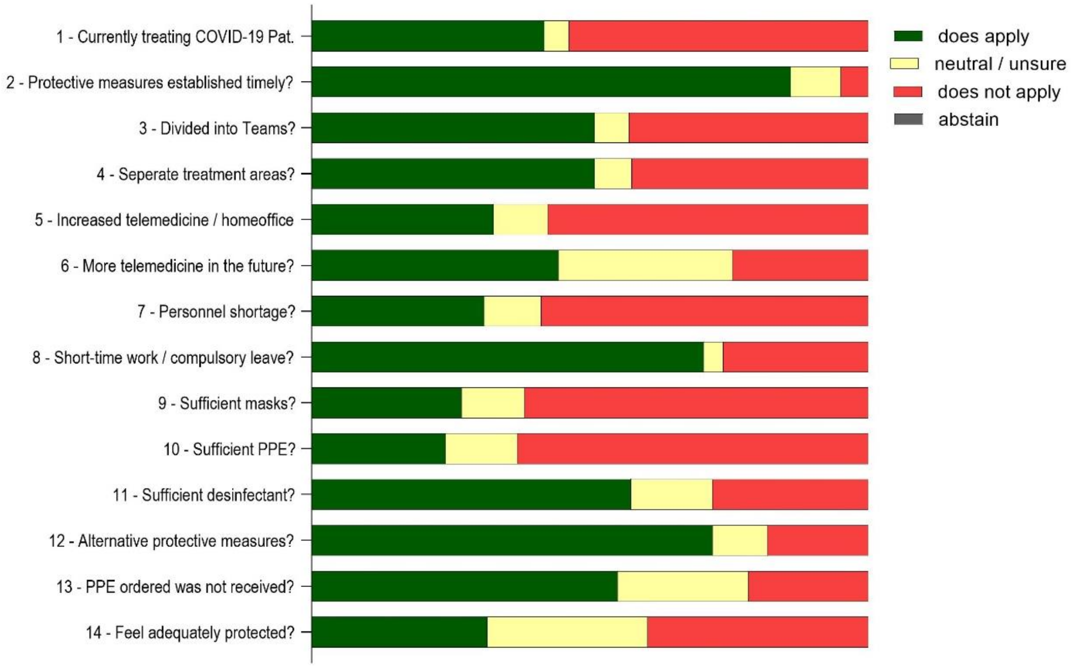

The general trend shows that the answers in these incomplete questionnaires are remarkably like those that were analyzed for the manuscript. Almost all incomplete data sets were missing the demographics part of the survey, since these questions were asked last in the online questionnaire.

3. Second survey for demographics

Using a Google Form Document[2], we sent an independent second survey on May 28th and May 29th, 2020, to be completed anonymously and including only 6 questions concerning demographic items. The questions were equal to the last 6 items of the original survey (employment, position, patient insurance status, specialization, age, gender). Recipients were colleagues registered as orthopedics or trauma surgeons in the greater area around Bonn, who frequently refer patients to our department. The mailing list was therefore distinct from that used for the primary survey. By May 29th, 2020, 6:00 pm, 52 participants had completed the form:

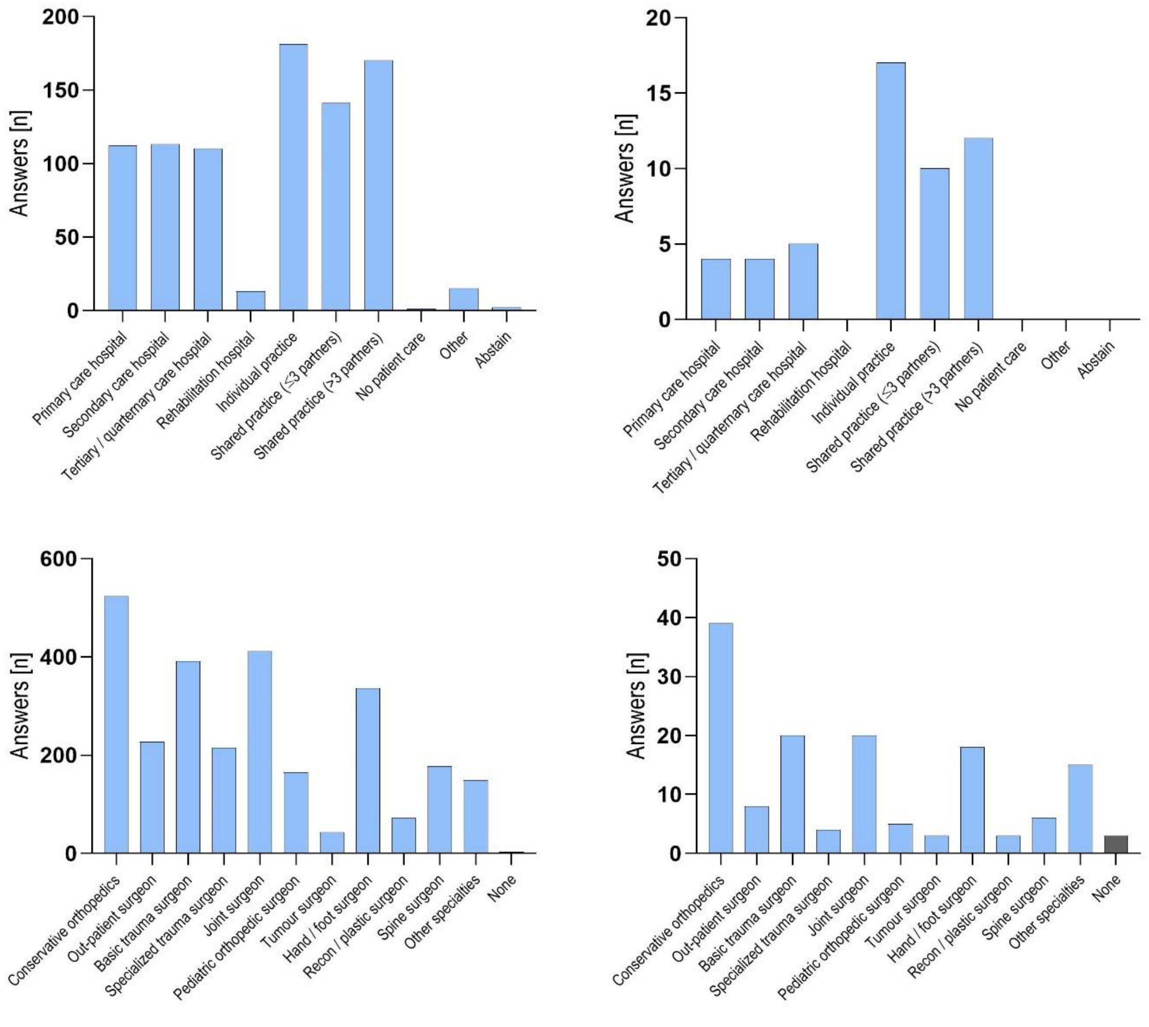

[2] https://docs.google.com/forms/d/e/1FAIpQLSc5xCifNjDq7dQzepFQbU9CbWb7vKYKYFthunhjvnTd62QZKQ/viewform?vc=0&c=0&w=1

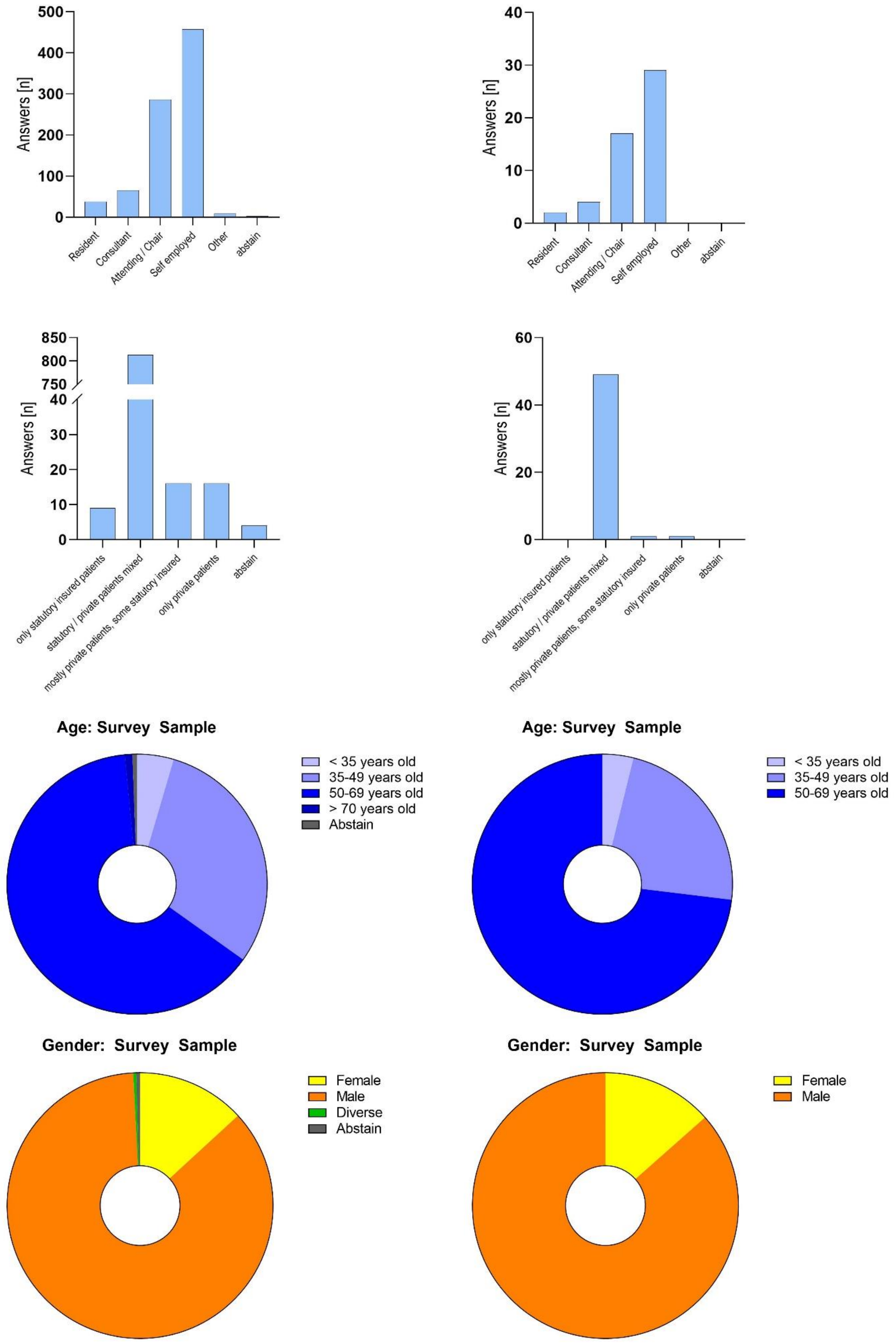

The left-hand graphs depict the results from the original survey (858 participants) as published in the manuscript in Figure 2. The right-hand graphs are the results of the demographic mini-survey. The results are, overall, very similar, and we interpret this that our collective is representative to orthopedics and trauma surgeon. The bias of “online survey”, however, remains, and we cannot guarantee that there is no overlap between the two collectives, since both surveys were conducted anonymously.
