# Supplementary material for: Collateral effect of COVID-19 on orthopedic and trauma surgery
Source: PLoS One. 2020 Sep 8;15(9):e0238759. doi: 10.1371/journal.pone.0238759 (PMC7478708; doi:10.1371/journal.pone.0238759)
Supplement: Attachment — Submitted filename: Rebuttal_Letter.docx [file pone.0238759.s008.docx]

Staff Editors

PLOS

Berlin, 18^th^ of June 2020

Dear editors of PLOS one,

Thank you for your review of our manuscript and the helpful comments of the reviewers. Herewith, we are submitting the revised version of our manuscript entitled

**Perceived Impact of the COVID-19 Pandemic on Orthopaedic and Trauma Surgery in Germany – Implications for Future Crises in a nationwide Survey**

We have addressed all comment of the editorial office and the reviewers, and hereby submit our point-by-point reply:

1. Please ensure that your manuscript meets PLOS ONE's style requirements, including those for file naming.

*We have checked the style requirements and updated the style of the manuscript as needed.*

2. Thank you for stating in the text of your manuscript "The study protocol was reviewed and approved by the institutional ethics board (Approval No. #20/127)". Please also add this information to your ethics statement in the online submission form. In addition, in your ethics statement please state that this was an online anonymous survey. a. Please amend your current ethics statement to include the full name of the ethics committee/institutional review board(s) that approved your specific study. b. Once you have amended this in the Methods section of the manuscript, please add the same text to the “Ethics Statement” field of the submission form (via “Edit Submission”)

*We have included the ethics statement in the materials section of the manuscript, as well as completed the ethics statement in the online submission as required*

3. Please include a summary table of any collected demographic data.

*No other demographic data than those already displayed in Figure 2 was collected. We therefore do not see any additional value in also providing this data as a table. However, if needed, the data can be included, we have uploaded it as supplemental Table 2 in the online submission system.*

4. Please include a separate caption for each figure in your manuscript.

*We have included figure legends to figures 1-4 in the manuscript.*

5. Please remove any funding-related text from the manuscript

*Done*

5. ..let us know how you would like to update your Funding Statement. Currently, your Funding Statement reads as follows:

[The author(s) received no specific funding for this work.]

*Still true. No adjustments necessary.*

Additionally, because some of your funding information pertains to commercial funding, we ask you to provide an updated Competing Interests statement, declaring all sources of commercial funding. Please include the updated Competing Interests Statement and Funding Statement in your cover letter. We will change the online submission form on your behalf.

*None of the funding received by individual authors pertains any competing interest related to the study. No commercial funding was received in relation to the study. We have deleted the competing interest section within the manuscript.*

The development of the COVID-19 pandemia is nowadays well known and spread througout the media worldwide. Due to this background i would suggest to shorten the Introduction and focus on the development within germany as the base of your survey.

*Only the first paragraph of 123 words relates to the pandemia in general, the rest of the introduction is already focused on the german development. We have further shortened the introduction to address the reviewers concerns.*

The description of Table 1 is not fully conclusive, please reformulate clearer: "Descriptive analysis and data quality, as assessed by N, mean, standard deviation (SD), standard error of the mean (SEM), 95% confidence interval (95% CI), skewness, and kurtosis of all items where these analyses was sensibly possible."

*We have modified Table 1 description to be clearer in its’ meaning: “SD, SEM and 95% CI was calculated only for those items measured on the 5-degree scale or Likert skale, as well as for the calculated indices. For dichotome or multiselect items, these calculations are not sensibly possible”.*

Reading your manuscript i was wondering if there is a positive correlation between the level of preparedness, resources, informedness and existing concerns. A further analysis of this context might lead to a better flow of information limiting concerns in the future.

*Figure 3 holds the information the reviewer is asking for, though hard to recognize. The lower right corner of the figure presents a correlation matrix between the calculated indices. It shows that “concern” (last line but one in the matrix) correlates strongly negative with “resources” and “informedness”, and positively with “depletion”, less positively also with “reduction”. On Page 10, “Correlations and regression”, we are pointing this out in the text. However, we agree that this point is worth pointing out in regard to future handling, and therefore now have picked this up in the discussions section: “We could show that the index “concern” had a strong negative correlation with the indices “informedness” and “resources”, so in future handling of the crisis, information and resources are the key factors to diminish healthcare professionals’ anxiety.”*

Please provide full text before using the abbreviation “PPE”

*The abbreviation was defined inthe abstract, as requested.*

As only about half of the opened questionnaires have been returned, it would be interesting to know whether the sample analyzed was representative. Do you have demographic data of the unreturned questionnaires or could you estimate these numbers based on numbers of hospital and outpatient trauma/orthopaedic surgeons in Germany?

*We have completed an analysis of the incomplete surveys, and have attached this to the submitted revision. The PDF “SupplData3_ Sample Size and selction bias” addresses the reviewers very good question. If needed, the data can also be included in the final manuscript as supplementary material.*

Supplementary Material (A) and (B) with the original questionnaire are missing.

*We have included the original and the translated questionnaire (originally referenced as “A”) as supplementary data 1 and 2 in the resubmission, as well as the table of index questions (originally “B”) as SupplTable1_Index questions.*

Whereas the index questions are clearly described in the methods part, the different groups of surgeons (independent profile variables) are not mentioned. Please add this to the methods part. Partially the information can be found in Figure 2. However, it is not congruent to the variables used in Table 2.

*With appending the original survey, the different groups of surgeons and professional levels are clearly defined. We see this as sufficient for the reader, and have added the original and translated survey as intended. Table 2 only lists those independent profile variables that were significant in the bivariate analysis, and is therefore not congruent with the figure 2, which is a mere summary of the answers given.*

It would be informative to have a “Table 1“ giving crude numbers of baseline data for the different independent profile variables (hospital employed, working in a small unit etc) and each index question.

*In this case we do not agree with the reviewer. Figure 1 and 2 are a graphical representation of the numbers, as the answers were given by the participants, an additional table with the numbers would not add any information to this. Should the editor decide that a table with numbers is beneficial rather than only figures, we can provide this.*

I am not sure whether the many numbers in Table 1 are easy to understand for the reader. It would be helpful to have an example for at least one predictor explained in text. E.g. “Reduction in outpatient clinic with a mean of 0.7673 means that …”

*We have added a paragraph to the results section, explaining how Likert scales transform into numerical values to be statistically analyzed, and how to grade this. We abstain from explaining Skewness, Kurtosis and other statistical measures, assuming the statistically inclined reader will be familiar and able to interpret.*

In general, the manuscript is methodologically sound, but very “technical” and therefore not easy to understand for the reader. I suggest to add legends for Figures 3 and Suppl. Figure 1, each containing an example that explains how to read the diagrams.

*We have added figure legends (see above), and included an example as suggested in Figure legend 3.*

We thereby see all issues raised by the reviewers as addressed and would very much appreciate our manuscript to be considered for publication.

Sincerely

Matthias Pumberger	Thomas Randau

For the Authors
